# Enhanced macrophage delivery to the colon using magnetic lipoplexes with a magnetic field

**DOI:** 10.1080/10717544.2019.1662515

**Published:** 2019-09-18

**Authors:** Yusuke Kono, Serika Gogatsubo, Takeshi Ohba, Takuya Fujita

**Affiliations:** a Ritsumeikan-Global Innovation Research Organization, Ritsumeikan University, Kusatsu, Japan;; b Laboratory of Molecular Pharmacokinetics, College of Pharmaceutical Sciences, Ritsumeikan University, Kusatsu, Japan;; c Research Center for Drug Discovery and Development, Ritsumeikan University, Kusatsu, Japan

**Keywords:** Cell delivery, magnetic lipoplex, magnetic field, macrophage, colon delivery, plasmid DNA

## Abstract

Magnetically guided cell delivery systems would be valuable to achieve effective macrophage-based cell therapy for colonic inflammatory diseases. In the current study, we developed a method for the efficient and simultaneous introduction of superparamagnetic iron oxide nanoparticles (SPIONs) and plasmid DNA (pDNA) into RAW264 murine macrophage-like cells using SPION-incorporated cationic liposome/pDNA complexes (magnetic lipoplexes). SPIONs and pDNA were introduced for magnetization and functionalization of the macrophages, respectively. We also evaluated the adhesive properties of magnetized RAW264 cells using magnetic lipoplexes in the murine colon under a magnetic field. Significant cellular association and gene expression without cytotoxicity were observed when magnetic cationic liposomes and pDNA were mixed at a weight ratio of 10:1, and SPION concentration and magnetic field exposure time was 0.1 mg/mL and 10 min, respectively. We also observed that cytokine production in magnetized RAW264 cells was similar to that in non-treated RAW264 cells, whereas nitric oxide production was significantly increased in magnetized RAW264 cells. Furthermore, magnetized RAW264 cells highly adhered to a Caco-2 cell monolayer and colon in mice, under a magnetic field. These results suggest that this magnetic cell delivery system can improve the colonic delivery of macrophages and its therapeutic efficacy against colonic inflammatory diseases.

## 1. Introduction

Macrophages demonstrate diversity and plasticity in their immune responses, and they alter their phenotype in response to the surrounding environment (Sica & Mantovani, 2012; Mantovani et al., 2013; Parisi et al., 2018). Based on their phenotype, macrophages have a bifunctional nature that promotes or suppresses the progression of several colonic inflammatory diseases, including inflammatory bowel disease (IBD) and colorectal cancer. Classically activated (M1) macrophages are characterized by an interleukin (IL)-10^low^ and IL-12^high^ phenotype. They produce a lot of inflammatory cytokines, including TNF-α, IL-6, and IL-1β, contributing to epithelial apoptosis, barrier dysfunction, and necrosis in active IBD (Heinsbroek & Gordon, 2009; Steinbach & Plevy, 2014). However, M1 macrophages have also been known to contribute to the inhibition of tumor progression (Yuan et al., 2015; Guo et al., 2016). In contrast to M1 macrophages, alternatively activated (M2) macrophages exhibit an IL-10^high^ and IL-12^low^ phenotype. These M2 macrophages enhance colonic cancer progression and metastasis by secreting various growth factors, including vascular endothelial growth factor, fibroblast growth factor, and matrix metalloproteases (Yuan et al., 2015; Yang & Zhang, 2017). M2 macrophages also have the potential to suppress colitis via the expression of anti-inflammatory cytokines, such as IL-10 and transforming growth factor-β (Weisser et al., 2011; Cheng et al., 2017). The delivery of appropriately polarized macrophages into the colon could be a promising therapy for colonic inflammation-related diseases. A previous study has shown that the transplantation of M1 macrophages into tumors inhibited tumor growth (Yeung et al., 2015). In addition, Hunter et al. (2010) demonstrated that in-vitro-derived M2 macrophages significantly reduced the severity of murine colitis.

To achieve effective macrophage-based cell therapy for colonic inflammatory diseases, the efficient colonic delivery and retention of macrophages are needed. A magnetic approach has been considered to be a valuable method for targeted cell delivery (Kobayashi et al., 2008; Polyak et al., 2008; Moysidis et al., 2015). This method consists of a two-step process: the introduction of magnetic nanoparticles into the cells, and the magnetic guidance of the magnetized cells through site-specific exposure to an external magnetic field. It has been reported that superparamagnetic iron oxide nanoparticle (SPION)-loaded mesenchymal stem cells have a high capacity to adhere to and remain in targeted damaged tissues under an external magnetic force (Kobayashi et al., 2008). Other groups have also demonstrated that SPION-loaded endothelial cells can be drawn toward a magnetic field, and these cells are available for reendothelialization in blood vessels and the cornea (Polyak et al., 2008; Moysidis et al., 2015). These findings prompted us to develop a magnetic targeting system for macrophages in the colon.

For the efficient introduction of SPIONs into macrophages, we intended to exploit cationic liposomes, because we have previously prepared cationic liposomes with encapsulated SPIONs (magnetic cationic liposomes) and demonstrated that they underwent cellular uptake in a short time under a magnetic field (Kono et al., 2017). In addition, cationic liposomes can form a complex with plasmid DNA (pDNA) (Masotti et al., 2009), and therefore, magnetic cationic liposomes would enable the simultaneous delivery of SPIONs and pDNA into macrophages. pDNA transfection is valuable for the functionalization of macrophages and the regulation of their polarization (Tran et al., 2015; He et al., 2018).

Here, we prepared magnetic cationic liposome/pDNA complexes (magnetic lipoplexes), and evaluated their uptake and gene transfection efficiency in macrophages. In addition, the adhesive properties of magnetic lipoplex-loaded macrophages (magnetized macrophages) under a magnetic field were also investigated both *in vitro* and *in vivo*.

## 2. Materials and methods

### 2.1. Ethics statement

All animal experiments were carried out in accordance with the Guide for the Care and Use of Laboratory Animals, as adopted and promulgated by the U.S. National Institutes of Health (Bethesda, MD, USA) and the Guidelines for Animal Experiments of Ritsumeikan University (Shiga, Japan). The protocol was approved by Ritsumeikan University Animal Experimentation Committee (approval number: BKC2014-003). All efforts were made to minimize suffering.

### 2.2. Animals and cell lines

Female ICR mice (4 weeks old) were purchased from SLC, Inc. (Shizuoka, Japan).

RAW264 murine macrophage-like cells were provided by the RIKEN BioResource Center Cell Bank (Ibaraki, Japan). Caco-2 human epithelial colorectal adenocarcinoma cells were purchased from DS Pharm Biomedical Co., Ltd (Osaka, Japan). RAW264 cells and Caco-2 cells were cultured in RPMI-1640 medium and Dulbecco’s Modified Eagle Medium, respectively, supplemented with 10% heat-inactivated fetal bovine serum, penicillin G (100 U/mL), and streptomycin (100 µg/mL) at 37 °C in 5% CO_2_/95% air.

### 2.3. Construction of magnetic lipoplexes

Magnetic cationic liposomes were prepared as reported previously (Kono et al., 2017). Magnetic cationic liposomes consisted of 1,2-dioleoyl-3-trimethylammonium-propane (DOTAP) (Avanti Polar Lipids Inc., Alabaster, AL, USA), 1,2-distearoyl-*sn*-glycero-3-phosphocholine (DSPC) (Avanti Polar Lipids), and cholesterol (Nacalai Tesque, Kyoto, Japan) at molar ratios of 5:0:5, 4:1:5, 3:2:5, or 2:3:5. All liposomes were labeled with 1 mol% (N-(Fluorescein-5-thiocarbamoyl)-1,2-dihexadecanoyl-*sn*-glycero-3-phosphoethanolamine (Thermo Fisher Scientific K.K., Kanagawa, Japan). In brief, the lipids were dissolved in chloroform, and a lipid film was formed through the evaporation of the solvent. After vacuum desiccation, magnetic cationic liposomes were produced through hydration of the lipid film with a 5% glucose solution containing 0.1 mg/mL or 0.5 mg/mL of iron oxide (II, III) magnetic nanoparticles (Sigma-Aldrich, St. Louis, MO, USA) under mechanical agitation. The liposomes were purified through centrifugal filtration using Amicon^®^ Ultra Centrifugal Filters (MWCO: 100,000; Merck Millipore, Tokyo, Japan). The encapsulation efficiency of the magnetic nanoparticles in the liposomes was approximately 16%. The lipoplexes were constructed by gently mixing magnetic cationic liposomes with a pGL4.50 [luc2/CMV/Hygro] vector (Promega, Madison, WI, USA) at weight ratios of 5:1, 10:1, 20:1, 50:1, or 100:1, with the resultant mixture being incubated for 30 min at room temperature. The particle sizes and ξ-potentials of the magnetic lipoplexes were measured using a Zetasizer Nano ZS instrument (Malvern Instruments, Worcestershire, UK).

### 2.4. Cellular association and/or uptake experiments

RAW264 cells were plated in 24-well culture plates at a density of 2 × 10^5^ cells/cm^2^, and cultured for 24 h. The cells were washed using phosphate buffered saline (PBS), and placed on a magnetic plate (OZ Biosciences, San Diego, CA, USA). The size of the magnetic plate was 8 cm × 12 cm, 1 cm thickness. The magnetic field intensity was approximately 300 mT. Magnetic lipoplexes (containing 1 µg of pDNA) in Opti-MEM I (Thermo Fisher Scientific K.K.) were added to the well, and incubated for predetermined times (5, 10, 30, 60, and 120 min). Then, the cells were washed twice using ice-cold PBS and lysed using lysis buffer (0.05% Triton X-100, 2 mM ethylenediaminetetraacetic acid, 0.1 M Tris, pH 7.8). After centrifugation at 10,000 × *g* for 10 min at 4 °C, the amount of magnetic cationic liposomes in the supernatant was quantified by measuring the fluorescence intensity using an Infinite F200 microplate reader (Tecan Japan Co., Ltd, Kanagawa, Japan).

### 2.5. Cell viability assay

RAW264 cells were plated in 24-well culture plates at a density of 2 × 10^5^ cells/cm^2^, and cultured for 24 h. The cells were washed using PBS, and placed on a magnetic plate (OZ Biosciences). Magnetic lipoplexes (containing 1 µg of pDNA) in Opti-MEM I (Thermo Fisher Scientific K.K.) were added to the well, and incubated for a predetermined time. After the incubation, the medium was replaced with fresh RPMI-1640 medium, and the cells were incubated for an additional 24 h at 37 °C in 5% CO_2_/95% air. Then, cell viability was measured using Cell Counting Reagent SF (Nacalai Tesque) and an Infinite F200 microplate reader (Tecan Japan Co., Ltd.). The results were expressed as viability (%).

### 2.6. Measurement of luciferase activity

After the cell viability assay, the cells were lysed using lysis buffer. After centrifugation at 10,000 × *g* for 10 min at 4 °C, the supernatant was mixed with Picagene LT 2.0 luminescence reagent (Toyo Ink, Tokyo, Japan), and incubated for 30 min. Luciferase activity was measured using an SH-8100 microplate reader (Corona Electric Co., Ltd, Ibaraki, Japan).

### 2.7. Measurement of cytokine and nitric oxide production from macrophages

RAW264 cells were plated in 24-well culture plates at a density of 2 × 10^5^ cells/cm^2^, and cultured for 24 h. The cells were washed using PBS, and were treated using magnetic lipoplexes (weight ratio of magnetic cationic liposomes:pDNA = 10:1, for each 1 µg of pDNA) for 10 min under a magnetic field. Then, the medium was replaced using fresh RPMI-1640 medium with or without lipopolysaccharide (LPS) (100 ng/mL), and incubated at 37 °C in 5% CO_2_/95% air. At 24 h after treatment with the magnetic lipoplexes, the supernatants were collected. The concentrations of IL-6, IL-10, IL-12, and TNF-α in the supernatants were measured using commercial ELISA kits (PeproTech Inc., Rocky Hill, NJ, USA). Nitric oxide (NO) concentrations in the supernatants were measured using a Griess assay, as previously reported (Kono et al., 2016).

### 2.8. *In vitro* adhesion of macrophages to a caco-2 cell monolayer

To prepare a Caco-2 cell monolayer, Caco-2 cells were seeded in a 12-well culture plate at a density of 5 × 10^4^ cells/cm^2^, and cultured for 14 days. RAW264 cells were seeded in a 10-cm culture dish at a density of 2 × 10^5^ cells/cm^2^, and cultured for 24 h. RAW264 cells were treated using magnetic lipoplexes (weight ratio of magnetic cationic liposomes:pDNA = 10:1) for 10 min under a magnetic field, and incubated for 6 h at 37 °C in 5% CO_2_/95% air. Then, magnetized RAW264 cells were fluorescently labeled using Calcein-AM (Nacalai Tesque), washed twice using PBS, and collected through trypsinization. For an upright positioned study, a 12-well culture plate with Caco-2 cell monolayers was placed on a magnetic plate. Magnetized RAW264 cells were added to the wells at a density of 1 × 10^5^ cells/cm^2^, and incubated for 5 min with or without a magnetic field. For an inverted positioned study, wells containing Caco-2 cells were filled with RPMI-1640 medium, and tightly sealed. Then, the plate was reversed, and placed under a magnetic field. Magnetized RAW264 cells were added to the wells at a density of 1 × 10^6^ cells/cm^2^ using a syringe with a 26-gauge needle (TERUMO Corporation., Tokyo, Japan), and incubated for 30 min with or without a magnetic field. After washing twice using PBS, the adherent RAW264 cells were observed using a fluorescence BZ-X710 microscope (KEYENCE Corporation, Tokyo, Japan).

### 2.9. *In vivo* adhesion assay of macrophages in mouse colon

ICR mice were fasted for 12 h prior to and during the experiment. The mice were anesthetized through isoflurane inhalation, and were intrarectally injected with 1 × 10^6^ magnetized RAW264 cells. Immediately after the cell injection, a magnetic field was applied to the abdominal area for 1 h. After the injection (6 h or 24 h), the mice were sacrificed and their colons were collected, rinsed using PBS, and weighed. The colons were homogenized using lysis buffer, and centrifuged at 10,000 × *g* for 10 min at 4 °C. The luciferase activity in the supernatant was measured using an SH-8100 microplate reader (Corona Electric Co., Ltd). To convert the luciferase activity to the number of magnetized RAW264 cells, together with the colon samples, 1 × 10^4^, 2 × 10^4^, 5 × 10^4^, 1 × 10^5^, 2 × 10^5^, and 5 × 10^5^ cells of magnetized RAW264 cells were also homogenized, centrifuged, and the luciferase activity in the supernatant was measured. Then, the regression line was made, and the number of cells was determined by applying the luciferase activity of colon samples to the regression line.

### 2.10. Statistical analysis

The results are presented as the mean + standard deviation (SD) of four experiments. Analysis of variance (ANOVA) was used to test the statistical significance of differences between groups. Two-group comparisons were performed using Student’s *t* test. Multiple comparisons between control groups and other groups were performed using Dunnett’s test.

## 3. Results

### 3.1. Optimization of the lipid composition of the magnetic cationic liposomes

**Figure 1.**
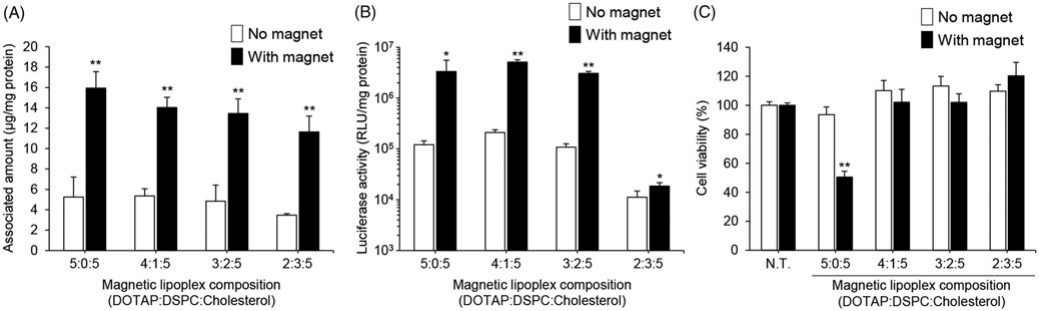
Effect of the lipid composition of the magnetic cationic liposomes on their cellular association, pDNA transfection efficiency, and cytotoxicity in RAW264 cells. Cellular association and/or uptake of magnetic cationic liposomes (A) and the level of luciferase expression following magnetic lipoplex administration (B). Magnetic lipoplexes prepared using various lipid compositions (DOTAP:DSPC:cholesterol = 5:0:5, 4:1:5, 3:2:5, or 2:3:5) and containing 0.1 mg/mL of SPIONs were added to each well (as 1 µg of pDNA), and incubated for 10 min at 37 °C in the presence or absence of a magnetic field. The mixing ratio of the magnetic cationic liposomes/pDNA was 10:1. Each value represents the mean + SD (*n* = 4). *p < .05; **p < .01, compared with no magnet. (C) Cell viability of RAW264 cells incubated with magnetic lipoplexes (as 1 µg of pDNA) for 10 min at 37 °C in the presence or absence of a magnetic field. Each value represents the mean + SD (*n* = 4). **p < .01, compared with non-treated (N.T).

To obtain optimal cell uptake and transfection efficiency in RAW264 cells without cytotoxicity, we evaluated the effect of the lipid composition of the magnetic cationic liposomes on their cellular uptake, pDNA transfection efficiency, and cytotoxicity. The physicochemical properties of the magnetic lipoplexes are listed in Table 1. The particle sizes of the magnetic lipoplexes were in the range of 620 nm to 680 nm. Their ζ-potential increased as the ratio of DOTAP in the liposomes increased. In addition, we confirmed the formation of the magnetic lipoplexes by gel electrophoresis (Supplementary Figure S1). The migration of pDNA was not detected in the magnetic lipoplexes, indicating that the pDNA was successfully loaded onto the magnetic cationic liposomes. As shown in Figure 1(A), the amount of cell-associated magnetic cationic liposomes was significantly increased by the presence of a magnetic field. In addition, the level of cell-associated liposomes gradually decreased as the DOTAP ratio in the liposomes decreased. Moreover, the luciferase expression levels obtained using magnetic lipoplexes at a molar ratio of 5:0:5, 4:1:5, and 3:2:5 (DOTAP:DSPC:cholesterol) were much higher than those obtained using magnetic lipoplexes at a molar ratio of 2:3:5 (Figure 1(B)). We also investigated the cell viability of RAW264 cells treated using magnetic lipoplexes. A significant reduction in cell viability was observed with magnetic lipoplexes at a molar ratio of 5:0:5, whereas the other magnetic lipoplexes produced no cytotoxicity (Figure 1(C)). Based on these results, we decided that the optimal lipid composition for the magnetic cationic liposomes is DOTAP:DSPC:cholesterol at a molar ratio of 4:1:5.

**Table 1.** Particle sizes and ζ-potentials of magnetic lipoplexes.

Lipid composition (mol)		
(DOTAP:DSPC:cholesterol)	Particle size (nm)	ζ-potential (mV)
5:0:5	675 ± 18	66.3 ± 9.3
4:1:5	622 ± 12	64.9 ± 8.9
3:2:5	641 ± 14	58.0 ± 11.4
2:3:5	648 ± 14	48.1 ± 13.7

The mixing ratio of the magnetic cationic liposomes/pDNA was 10:1.

Each value represents the mean ± SD (*n* = 4).

### 3.2. Optimization of the magnetic cationic liposome/pDNA mixing ratio

**Figure 2.**
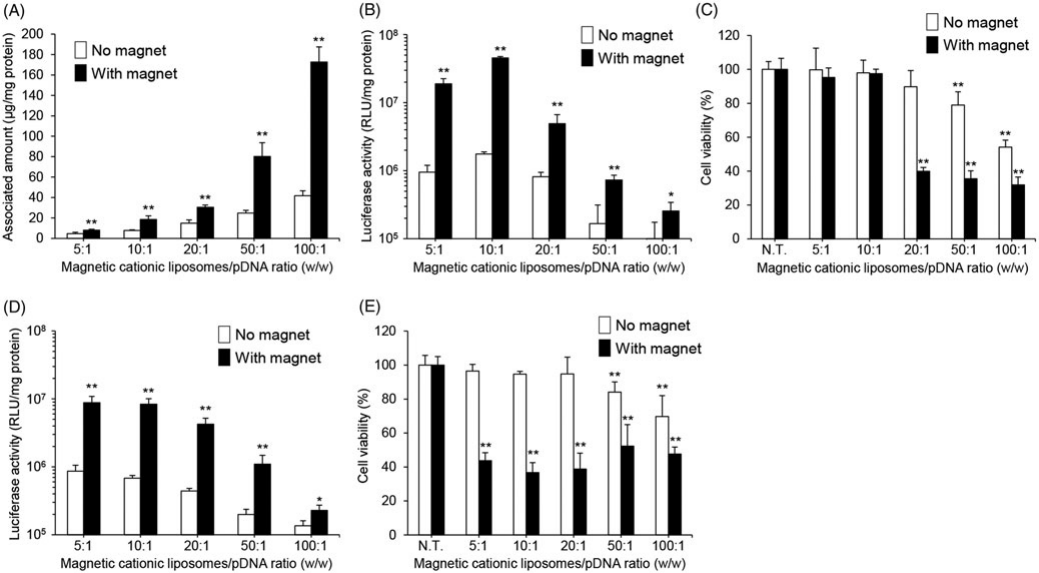
Effect of magnetic cationic liposome/pDNA ratio and SPION concentration on the cellular association, pDNA transfection efficiency, and cytotoxicity of magnetic lipoplexes in RAW264 cells. Cellular association and/or uptake of magnetic cationic liposomes containing 0.1 mg/mL of SPIONs (A) and the level of luciferase expression produced by magnetic lipoplexes containing 0.1 mg/mL (B) and 0.5 mg/mL (D) of SPIONs. Magnetic lipoplexes (DOTAP:DSPC:cholesterol = 4:1:5) were added to each well (as 1 µg of pDNA), and incubated for 10 min at 37 °C in the presence or absence of a magnetic field. Each value represents the mean + SD (*n* = 4). *p < .05; **p < .01, compared with no magnetic field. Cell viability of RAW264 cells incubated with magnetic lipoplexes containing 0.1 mg/mL (C) and 0.5 mg/mL (E) of SPIONs (as 1 µg of pDNA) for 10 min at 37 °C in the presence or absence of a magnetic field. Each value represents the mean + SD (*n* = 4). **p < .01, compared with N.T.

Using magnetic cationic liposomes with the optimized lipid composition, we next assessed the effect of the mixing ratio of the magnetic cationic liposomes to pDNA on their cellular uptake, transfection efficiency, and cytotoxicity. Here, we used two magnetic cationic liposomes with different SPION concentrations (0.1 and 0.5 mg/mL). In magnetic lipoplexes with 0.1 mg/mL of SPIONs, the cellular association of the magnetic cationic liposomes increased as the mixing ratio was increased (Figure 2(A)). In addition, the highest luciferase expression was obtained with the 10:1 mixing ratio (Figure 2(B)). Furthermore, cytotoxicity was not observed at the mixing ratios of 5:1 and 10:1, whereas a significant reduction in cell viability was observed with mixing ratios above 20:1 (Figure 2(C)). In the case of magnetic lipoplexes with 0.5 mg/mL of SPIONs, the luciferase expression profile was similar to that of magnetic lipoplexes with 0.1 mg/mL of SPIONs (Figure 2(D)). However, significant cytotoxicity was exhibited at all the mixing ratios (Figure 2(E)). Taken together, the mixing of magnetic cationic liposomes with 0.1 mg/mL of SPIONs and pDNA at a weight ratio of 10:1 is suitable for the efficient introduction of magnetic lipoplexes into RAW264 cells without cytotoxicity.

### 3.3. Optimization of magnetic field exposure time

**Figure 3.**
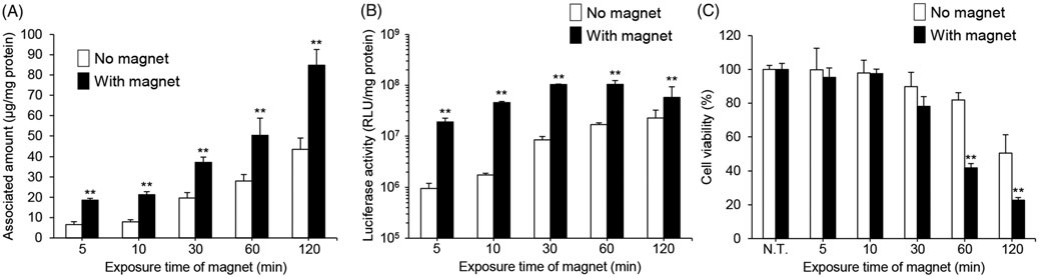
Effect of magnetic field exposure time on the cellular association, pDNA transfection efficiency, and cytotoxicity of magnetic lipoplexes in RAW264 cells. Cellular association and/or uptake of magnetic cationic liposomes (A) and the level of luciferase expression produced by magnetic lipoplexes (B). Magnetic lipoplexes (DOTAP:DSPC:cholesterol = 4:1:5) containing 0.1 mg/mL of SPIONs were added to each well (as 1 µg of pDNA), and incubated for 5–120 min at 37 °C in the presence or absence of a magnetic field. The mixing ratio of the magnetic cationic liposomes/pDNA was 10:1. Each value represents the mean + SD (*n* = 4). **p < .01, compared with no magnetic field. (B) Cell viability of RAW264 cells incubated with magnetic lipoplexes (as 1 µg of pDNA) for 5–120 min at 37 °C in the presence or absence of a magnetic field. Each value represents the mean + SD (*n* = 4). **p < .01, compared with N.T.

We also investigated the effect of the magnetic field exposure time on gene expression and cell viability in RAW264 cells treated using magnetic lipoplexes. As shown in Figure 3(A,B), the cellular association and luciferase expression level were increased with an increase in exposure time. However, a reduction in cell viability was observed when the magnetic field exposure exceeded 30 min (Figure 3B). These results indicate that the appropriate magnetic field exposure time is 10 min.

### 3.4. The effect of magnetic lipoplexes on cytokine and NO production in RAW264 cells

**Figure 4.**
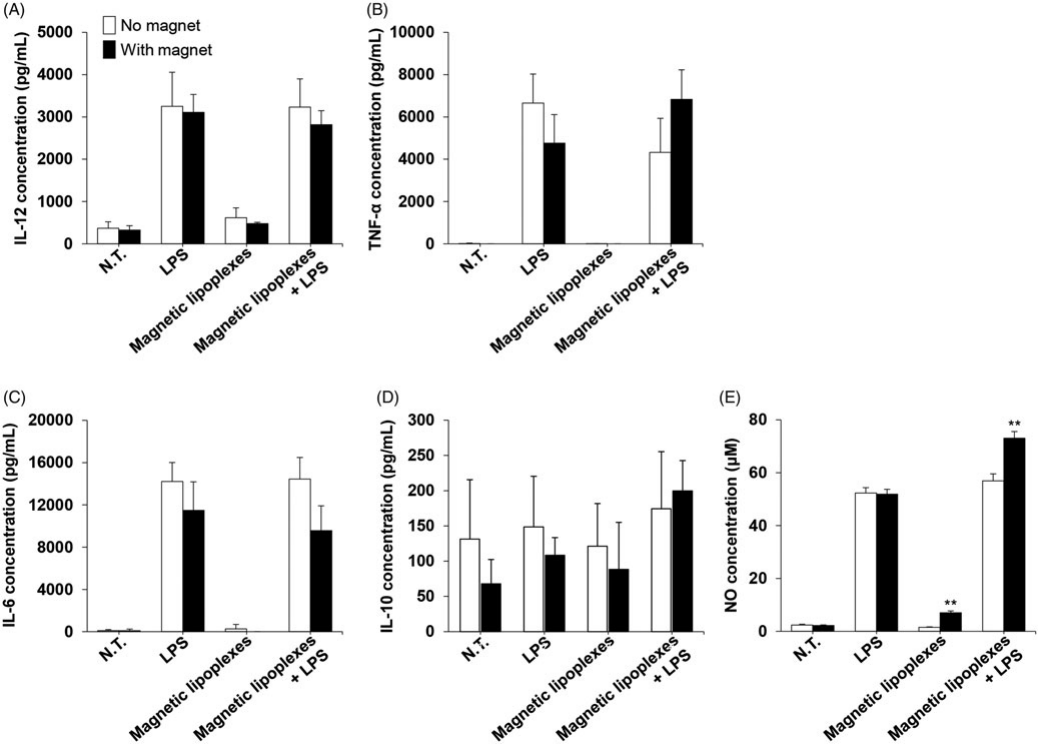
Cytokine and NO production in RAW264 cells treated with magnetic lipoplexes. Concentration of IL-12 (A), TNF-α (B), IL-6 (C), IL-10 (D), and NO (E) in the supernatant of RAW264 cells treated with or without magnetic lipoplexes (as 1 µg of pDNA) containing 0.1 mg/mL of SPIONs and LPS. The supernatants were collected 24 h after magnetic lipoplex treatment. Each value represents the mean + SD (*n* = 4). **p < .01, compared with N.T. macrophages.

We evaluated the immune response of magnetized RAW264 cells. IL-12, TNF-α, IL-6, and IL-10 production levels in RAW264 cells did not significantly change when magnetic lipoplexes were introduced (Figure 4(A–D)). In addition, cytokine production levels in both magnetized and nonmagnetized RAW264 cells were markedly increased by LPS stimulation. However, NO production in RAW264 cells was enhanced by the introduction of magnetic lipoplexes (Figure 4(E)).

### 3.5. Adhesion of magnetized RAW264 cells to caco-2 cell monolayers under a magnetic field

**Figure 5.**
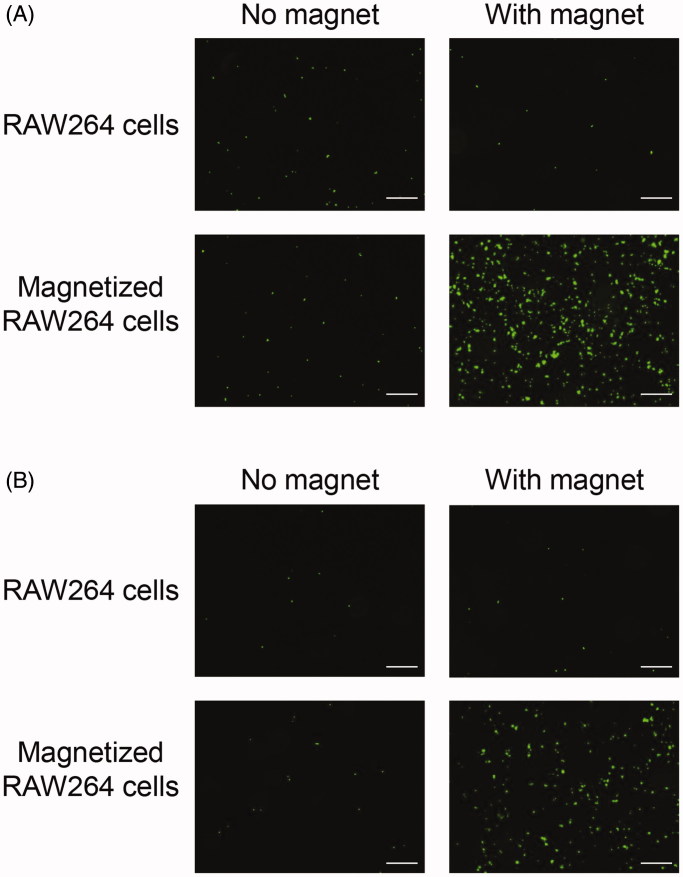
Adhesion of magnetized RAW264 cells to Caco-2 cell monolayers under a magnetic field. RAW264 cells treated with magnetic lipoplexes (DOTAP:DSPC:cholesterol = 4:1:5) containing 0.1 mg/mL of SPIONs were added to wells containing Caco-2 cell monolayers, and incubated for 5 min in an upright position (A) or for 30 min in an inverted position (B). RAW264 cells were fluorescently labeled with calcein-AM. Scale bar: 10 µm.

We investigated the adhesive properties of magnetized RAW264 cells in Caco-2 intestinal epithelial cell monolayers under a magnetic field. The number of magnetized RAW264 cells adhering to the monolayer was significantly increased under a magnetic field, and it was much higher than that of non-treated RAW264 cells (Figure 5(A)). We also carried out the same experiments under an inverted condition. Similar to the results of the experiment in an upright position, the number of adhering magnetized RAW264 cells under a magnetic field was markedly higher than without a magnetic field or with non-treated RAW264 cells (Figure (5B)).

### 3.6. *In vivo* delivery efficiency of magnetized RAW264 cells to the Colon under a magnetic field

**Figure 6.**
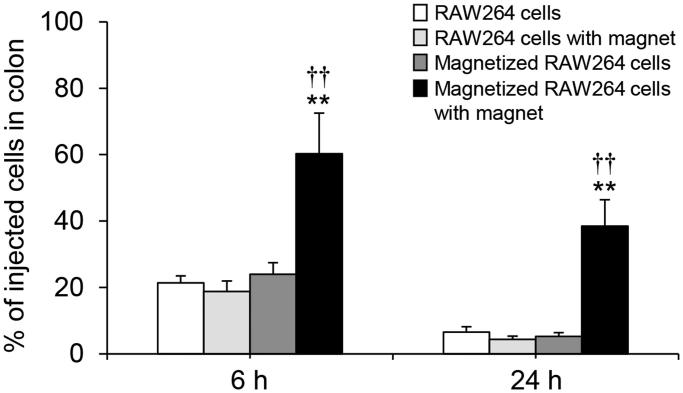
Adhesion of magnetized RAW264 cells to the colon under a magnetic field in mice. RAW264 cells treated with magnetic lipoplexes (DOTAP:DSPC:cholesterol = 4:1:5) containing 0.1 mg/mL of SPIONs were intrarectally injected into mice. The number of cells was measured at 6 and 24 h after the injection. Each value represents the mean + SD (*n* = 4). **p < .01, compared with RAW264 cells. ^††^*p* < .01, compared with magnetized RAW264 cells.

In addition to an *in vitro* adhesion assay, we assessed the adhesive efficiency of magnetized RAW264 cells in the colon under a magnetic field in mice. As shown in Figure 6, the number of adhering magnetized RAW264 cells in the colon was significantly increased in the presence of a magnetic field. Moreover, approximately 40% of the injected cells were retained in the colon until 24 h after the injection.

## 4. Discussion

In the current study, we describe an efficient method involving magnetic cationic liposomes for the simultaneous introduction of SPIONs and pDNA into macrophages. pDNA transfection has been reported as a useful method for polarizing and maintaining the phenotype of macrophages. He et al. (2018) demonstrated that the transfection of pDNA encoding the IL-12 gene into macrophages enables phenotype repolarization from M2 to M1. In addition, Jain et al. (2015) have shown that macrophages treated with pDNA encoding IL-10 were repolarized from M1 to M2. Therefore, magnetic lipoplexes delivering pDNA could enable the simultaneous magnetization of macrophages and regulation of their function.

Initially, we optimized the lipid composition of the magnetic cationic lipoplexes and the delivery protocol to obtain the highest cellular uptake efficiency for SPIONs and the highest pDNA transfection efficiency, without cytotoxicity. A positive charge is needed on the surface of liposomes to form complexes with pDNA and for efficient cell internalization, but cationic lipids are known to show high cytotoxicity (Lv et al., 2006; Masotti et al., 2009). We confirmed that magnetic lipoplexes composed of only DOTAP and cholesterol produced significant cytotoxicity in RAW264 cells (Figure 1(C)). However, the cytotoxicity of the magnetic lipoplexes was significantly attenuated by replacing some DOTAP with DSPC, a neutral phospholipid. The highest levels of liposome delivery and gene expression without cytotoxicity were observed with magnetic lipoplexes at a molar ratio of 4:1:5 (DOTAP:DSPC:cholesterol) (Figure 1(A,B)). On the other hand, the luciferase expression level obtained with the magnetic lipoplexes at a molar ratio of 3:2:5 was lower than that obtained with the other lipoplexes despite the fact that the cellular-associated quantities of all of the magnetic cationic liposomes were not significantly different. This may be due to the weaker interaction of the magnetic cationic liposomes with the pDNA. Although the pDNA was fully complexed with the magnetic cationic liposomes at a molar ratio of 3:2:5 after preparation (Supplementary Figure S1), the electrostatic interaction of pDNA with the liposomes would be weaker than that with other liposomes because of the lower surface charge. Therefore, pDNA may be released from the lipoplexes during the cellular internalization process, and consequently, pDNA would not reach the cytoplasm. Based on these findings, we determined this ratio to be the optimal lipid composition for the magnetic lipoplexes.

We also determined the optimal mixing ratio of the magnetic cationic liposomes with pDNA and the optimal concentration of SPIONs in the liposomes for efficient delivery of the magnetic lipoplexes into macrophages. The viability of RAW264 cells decreased in a liposome and SPION concentration-dependent manner (Figure 2(C,E)). It has been reported that SPIONs produce cytotoxicity in macrophages at a concentration of 6.25 μg/mL (Park et al., 2014), and the current magnetic lipoplexes contain SPIONs at approximately 0.06–1.2 μg/mL. Therefore, the cytotoxicity produced by the magnetic lipoplexes can be mainly attributed to the cationic lipids, not the SPIONs. While the cellular association of the magnetic cationic liposomes increased as the mixing ratio was increased, the highest luciferase expression was observed when the magnetic cationic liposomes were mixed with pDNA at a weight ratio of 10:1 (Figure 2(A,B)). It has been reported that a large amount of cationic liposomes tightly interact with pDNA, resulting in difficulty in releasing pDNA in the cytoplasm and subsequently limiting gene expression (Sakurai et al., 2000). Our results agree with this finding. Furthermore, we observed that the cellular association, gene transfection efficiency, and cytotoxicity of the magnetic lipoplexes were increased with an increased magnetic field exposure time (Figure 3). Taking these results together, we decided that the optimal mixing ratio of magnetic cationic liposomes/pDNA, SPION concentration, and the magnetic field exposure time is 10:1, 0.1 mg/mL, and 10 min, respectively. Since previous reports have shown that it takes approximately 24 h to label macrophages with SPIONs (Muthana et al., 2008; Riou et al., 2013; Tong et al., 2016), the present method could be useful for rapid labeling of cells with SPIONs.

In macrophage-based cell therapy for inflammatory diseases, therapeutic efficiency depends on the macrophage immune response. It has been reported that SPIONs affect cytokine production in immune cells, including macrophages (Hsiao et al., 2008; Yeh et al., 2010). In addition, several reports have shown that SPIONs induce a phenotypic shift in macrophages from M2 to M1 (Laskar et al., 2013; Gu et al., 2019). Therefore, we evaluated the effect of magnetic lipoplexes on cytokine and NO production in RAW264 cells. Cytokine secretion (IL12, TNF-α, IL-6, and IL-10) did not significantly change following magnetic lipoplex administration (Figure 4(A–D)). These results indicate that the magnetic lipoplexes did not affect the phenotypic characteristics of the macrophages. However, NO production was significantly enhanced by the magnetic lipoplexes (Figure 4(E)). It has been demonstrated that intracellular iron concentrations are closely related to NO production in cells (Galleano et al., 2004), and SPIONs enhance NO production in macrophages (Hsiao et al., 2008; Yeh et al., 2010). The present findings are in agreement with these previous reports. Since the increase in the level of NO production in RAW264 cells following magnetic lipoplex administration is modest, this immunological change is likely negligible or results in only a very small change in the therapeutic potential of the macrophages.

Finally, we evaluated the adhesive efficiency of the magnetized RAW264 cells under a magnetic field. In these investigations, we used magnetized RAW264 cells which were collected at 6 h after the magnetic lipoplex administration because it has been reported that luciferase expression produced by lipoplexes in macrophages reaches a maximum at 6 h after transfection (Nakamura et al., 2009; Un et al., 2010). In addition, Gu et al. (2011) demonstrated that internalized SPION levels in RAW264.7 cells are not significantly decreased until 36 h after SPIONs are administered to the cells. Therefore, this time point is also likely relevant to intracellular SPION levels in RAW264 cells. A significant increase in the adhesion of magnetized RAW264 cells to Caco-2 cell monolayers was observed in upright and inverted positions in the presence of a magnetic field (Figure 5). These results strongly suggest that magnetized RAW264 cells could respond to an external magnetic field in the body. We also observed that magnetized RAW264 cells showed remarkable adhesion in the murine colon in the presence of a magnetic field (Figure 6). Moreover, the adhesive magnetized RAW264 cells were largely retained in the colon for 24 h, although the magnetic field was applied for just 1 h after the injection of the cells. These results suggest that the strong adhesion of the magnetized cells to the tissue on initial application of the magnetic field is crucial to obtaining subsequent high retention of the cells.

## 5. Conclusion

We successfully developed a method for the rapid magnetization and pDNA transfection of macrophages, using magnetic lipoplexes. In addition, we demonstrated that magnetized macrophages efficiently adhere to and are retained in the magnetic field-exposed murine colon. Our present findings represent a valuable contribution toward the development of an effective cell therapy using magnetic guidance.
